# Commentary: An Unusual Case of Leadless Pacemaker Implantation in a Patient with Wolff-Parkinson-White Syndrome: Just Because We Can, Does That Mean That We Should?

**DOI:** 10.19102/icrm.2017.080501

**Published:** 2017-05-15

**Authors:** Ashish A. Bhimani, Bruce S. Stambler


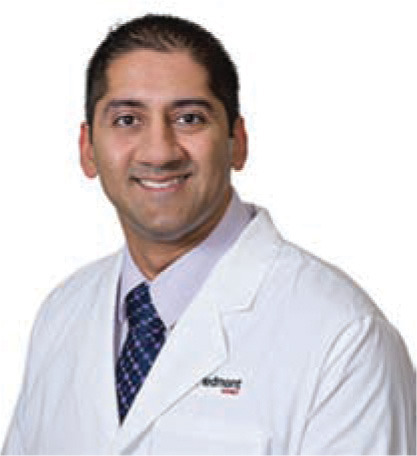


In the case presented by Drs. Tanaka-Esposito and Cantillon, a patient deemed to be at risk of developing unpredictable atrioventricular (AV) block based on electrophysiology findings and a catheter ablation procedure is chosen for the implantation of a leadless pacemaker that will provide permanent, prophylactic ventricular pacing. Briefly, this case involved a patient with a midseptal accessory pathway (AP) who had undergone a prior catheter ablation procedure for Wolff-Parkinson-White syndrome (WPW) 2 decades earlier. During the current procedure, anterograde AV conduction was seen over the accessory pathway with moderate (but not excessively rapid) conduction properties. A short application of low-energy radio-frequency (RF) current delivered in the midseptum transiently eliminated both pre-excitation and AV conduction. The authors concluded from these unexpected observations that antero-grade AV nodal-His-Purkinje conduction was markedly impaired (perhaps even absent), possibly due to permanent injury to the compact AV node incurred during the previous ablation procedure. This challenging and unusual case raises several questions that may have implications for clinical practice, including whether septal AP ablation remains a high-risk procedure; when to ablate or not ablate these APs; what is the natural history of unablated accessory pathways; and whether prophylactic pacemaker implantation was warranted in this case and, if so, what strategy would best provide the needed protection from symptomatic bradycardia


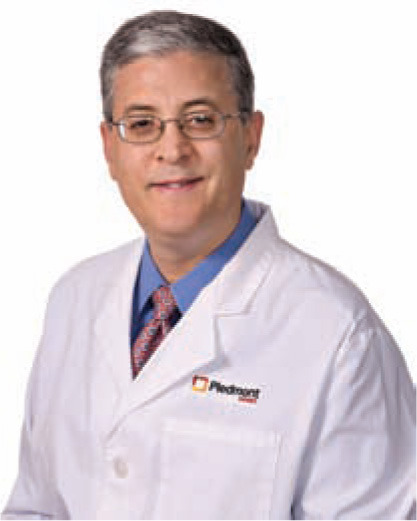


It may be controversial whether the accessory pathway should have been targeted for ablation in this case, given the absence of inducible tachycardia or malignant conduction properties indicative of a higher risk for sudden death (i.e. AP conduction at cycle lengths ≤250 ms), but it can be justified in our view, especially considering the patient-reported tachycardia symptoms. It is worth noting that successful ablation of an AP in the anteroseptal or midseptal region near the AV node-His-bundle conduction system can challenge the technical and intellectual skills of even the most experienced electrophysiologist. In the first few years after RF catheter ablation was introduced in the early 1990s (around the time of this patient’s first procedure), high rates of AV block were observed during septal AP ablation, with some operators recommending that the procedure be reserved only for patients with life-threatening or highly troublesome tachyarrhythmias.^1^ In 1996, it was reported that ablation of anteroseptal and midseptal APs in pediatric patients was associated with inadvertent AV block in 2.7% and 10.4% of cases, respectively, in comparison with posteroseptal APs, in which the AV block rate was 1%.^2^ However, recent ablation series have been much more reassuring, indicating that the risk of complete heart block currently is around 1% when ablating septal bypass tracts.^3^ Fortunately, the experiences of ablationists over the past few decades have taught that the elimination of AP conduction can be safely targeted and that permanent pacing can be avoided in virtually all cases. Ablationists have come to recognize that limiting the risk of permanent AV block with septal ablation requires a thorough understanding of septal anatomy, the payment of careful attention to catheter positioning and contact, consideration of low-power or alternative energy sources including cryoablation, and cautious monitoring of anterograde AV node-His-Purkinje conduction during energy delivery. Notably, the loss of manifest pre-excitation due to junctional ectopy during RF ablation should not be misinterpreted as elimination of AP conduction, but rather as heating of the compact AV node with the potential for imminent AV block. Pacing maneuvers, including parahisian or basal versus apical pacing, permit distinguishing AP from AV nodal conduction. Precisely localizing pathway potentials and differentiating these signals from His-bundle potentials is important in avoiding the impairment of AV nodal conduction during ablation in the septal region near the triangle of Koch.

Electrophysiologists caring for patients with WPW should maintain knowledge and understanding of the long-term, natural history of AP conduction in individuals who have not had their pathways successfully ablated. A recent study looked at a pediatric population with WPWand followed 133 children (ages eight to 12 years) for a median follow-up of 57 months, and found AP conduction did not spontaneously resolve in any of these patients.^4^ However, prior longitudinal studies, especially in adult cohorts with WPW, indicate that there is a substantial loss of AP conduction over time; although sample sizes were small (i.e. < 30 patients in each study), the rates of conduction loss varied from approximately 30% to as high as 78%.^5–7^ Predictors of the disappearance of pre-excitation are older age and longer effective refractory period.^5^ Thus, while APs in younger patients remain robust for a substantial period, the risk of loss of AP conduction is considerable over time in the adult population with WPW. In one center’s cohort study, among the 961 patients with pre-excitation included over a 25-year period, only 72 (7.5%) were aged between 60 and 85 (mean age 68.5 ± 6 years), and 26 patients were older than 69 (mean age 75 ± 17 years, 2.7%).^8^ Thus, one can counsel the patient presented in this current case that AP conduction loss is highly likely to occur over the next few decades of her life. However, when pre-excitation disappears, what is its time course (i.e. gradual or sudden)? Unfortunately, there are insufficient data on the progression speed of AP conduction loss. Whether the AP conduction worsens, either slowly resulting in progression from intermittent to complete loss of AP conduction or abruptly, can have an important clinical impact, especially when considering the proper management approach in the current case.

Despite the substantial risk for future loss of AP conduction in this patient, the absolute need for and timing of prophylactic pacing prior to the development of heart block is not definitive in this case. In this regard, it is reasonable to consider the adequacy of an escape rhythm in the presence of paroxysmal or complete AV block. Children born with congenital heart block are not under acute risk of bradycardia but have a gradual worsening of symptoms without the capacity to have sinus tachycardia as needed. If this patient lost her AP conduction, one might expect an adequate junctional rhythm to be sufficient for short-term safety, at which time a pacemaker could be implanted. However, given that she likely had prior ablation near the AVN-His-Purkinje region, any concerns about escape pacemaker foci being unreliable are certainly credible. A study evaluating escape rhythm adequacy after AV junction ablation (essentially those similar to this case) showed that 80% of patients had an adequate escape rhythm, and only 3 out of 40 patients lost their escape rhythm over 3-year follow-up.^9^ Although an implantable monitor could be considered as an alternative management approach in this patient, ultimately the likelihood of sudden heart block must be balanced against the risks of prophylactic pacemaker implantation. Given the relatively low risk of complications associated with pacemaker implantation as compared with the small but potentially serious possibility of sudden catastrophic heart block, the decision to proceed with pacemaker implantation is reasonable in this case.

What then is the optimal pacing system that should be implanted in this patient? Leadless pacemaker systems are a new disruptive technology that dramatically advances the options for pacemaker implants. When considering traditional transvenous pacemakers, some of their major risks include pocket hematoma, infection, and/or lead dislodgement or malfunction, all of which may be reduced by use of a leadless (and pocketless) device. Currently, the available leadless pacemaker systems are designed to replace single-lead right ventricular pacemakers (ventricle paced, ventricle sensed (VVI) or demand ventricular pacing with physiologic response to exercise (VVIR) pacing modes). There are currently two leadless pacemakers, the Nanostim Leadless Pacemaker (St. Jude Medical, Inc., St. Paul, MN) and the Micra Transcatheter Pacing System (Medtronic, Inc., Minneapolis, MN), both of which have been studied in major safety and efficacy trials.^10–13^ The Nanostim device (St. Jude Medical, Inc., St. Paul, MN, USA), which is Conformite Europeene (CE)-mark approved but not yet Food & Drug Administration (FDA) approved, is a cylindrical self-contained bipolar pacemaker, 42 mm in length with a diameter of 5.99 mm, delivered through an 18-Fr sheath. The device has a non-retractable single-turn screw at the distal tip, which is used for its attachment mechanism. The lithium carbon monofluoride battery is 248 mAh and is designed to last eight to 12 years. The Micra device (Medtronic, Inc., Minneapolis, MN, USA), which is both CE-mark and FDA-approved, is a cylindrical self-contained bipolar pacemaker, 25.9 mm in length with a diameter of 6.7 mm, that is delivered through a 23-Fr sheath. It has 4 distal nitinol tines that penetrate into the myocardium as its attachment mechanism. The lithium silver vanadium oxide/carbon monofluoride battery is 120 mAh and designed to last 5 to 10 years. Both devices have rate-responsive capabilities and have a docking knob for acute retrieval. In addition, though not studied prospectively, it is worth noting that both devices are expected to be magnetic resonance imaging safe due to a lack of ferrous material present in their designs.

The Nanostim device (St. Jude Medical, Inc., St. Paul, MN, USA) initially was evaluated in LEADLESS; a single-arm feasibility study in which 32 of 33 patients had a successful device implant, with two periprocedural complications, no late complications, and excellent performance measures in all successful implants.^1^ LEADLESS II was a single-arm safety and efficacy study that enrolled 526 patients who were followed for 6.9 ± 4.2 months.^13^ The pacemaker was successfully implanted in 504 patients (95.8%). There were 40 serious adverse events in 34 patients, for a total complication rate of 6.7%. The major complications were device dislodgement or migration (1.5%), cardiac perforation (1.6%), and vascular complications (1.2%). There were no reported infections. The efficacy results were assessed for 300 patients who completed six months of follow-up. Of this cohort, 289 patients underwent a successful implant, and 270 demonstrated good stable performance (defined as a capture threshold ≤ 2.0 V at 0.4 ms and a sensed R-wave ≥ 5.0 mV; both being stable over six months). Notably, the device was retrieved in seven patients, over a range of one to 413 days after implant.

The Micra (Medtronic, Inc., Minneapolis, MN, USA) has been studied in a safety and efficacy trial that followed 725 patients for a mean of four months.^10^ This pacemaker was successfully implanted in 719 patients (99.2%). There were 28 serious adverse events in 25 patients, for a total complication rate of 4.0%. The major complications were cardiac perforation (1.6%) and vascular complications (0.7%), with no gross dislodgements or infections. The efficacy results were assessed for 297 patients with a successful implant who reached six months of follow-up. Of this cohort, 292 showed stable performance (defined as an initial capture threshold ≤2.0 Vat 0.24 ms and an increase ≥1.5 Vover six months). The device was retrieved in one patient 17 days after implant.

As noted above, the two devices have a number of similarities, and their initial safety and efficacy results are quite comparable. However, there are important differences between devices that should be considered. Nanostim (St. Jude Medical, Inc., St. Paul, MN, USA) is a longer and narrower device with a larger overall volume (1.0 versus 0.8 cc). Nanostim’s (St. Jude Medical, Inc., St. Paul, MN, USA) battery size is substantially greater (248 mAh versus 120 mAh) than that of Micra (Medtronic, Inc., Minneapolis, MN, USA).^11^ Its narrower body also allows for a smaller access sheath, though vascular complications were found to be similar with both devices. Additionally, both devices’ attachment mechanisms are distinct, and Micra (Medtronic, Inc., Minneapolis, MN, USA) showed a high implant success rate with a much lower rate of dislodgement, though possibly at the expense of late device retrieval capability. The retrieval question may be an important one, particularly in the cases of younger patients. Among patients with a Nanostim implant (St. Jude Medical, Inc., St. Paul, MN, USA) who required chronic device retrieval (defined as occurring more than six weeks postimplant), 10 of 11 devices were successfully retrieved at a median duration of 220 days (88 to 1,188 days) postimplant without any retrieval-related complications.^14^

The efficacy of both devices appears to be reasonable, with generally good implant success and long-term electrical stability. The current acute complication rate is relatively high, but that may be partially due to the learning curve often present with the introduction of a new technology. There were a remarkably small number of late complications (albeit over a short follow-up period), and one would expect that as implant complications are reduced (via experience or revised techniques), the overall benefit of a leadless system would be enhanced. To date, the feasibility of leadless, right ventricular permanent pacing has been demonstrated in humans, providing proof-of-concept that this procedure can be done safely and effectively. Ultimately, prospective, longer-term studies of leadless pacemakers compared with conventional, transvenous pacing systems are needed, especially in broad, real-world practice settings. Thus, it is unknown whether the potential theoretical advantages of a leadless system will be realized versus existing transvenous options. Given the current implant-related complications, it would be hard to argue that the leadless pacemaker is currently a superior device, although the potential benefit of long-term risk reduction seems promising.

The patient in this case study has a few characteristics for which a leadless system might be theoretically preferred. She does not currently require pacing, so her expected pacing need is low in the near term, so there is no imminent need for a dual-lead system. She is relatively young, and preserving her upper extremity vasculature for any future needs (whether for implantation of another cardiac device or for dialysis access) has greater value. Another important consideration is battery life, given her age and the potential need for future devices after the initial battery is depleted. The Nanostim (St. Jude Medical, Inc., St. Paul, MN, USA) has a longer battery life due to its larger battery capacity. At the end of the battery life, the authors suggest a plan to implant a second device. There are data that the swine right ventricle can accommodate up to three Micra devices^15^ (Medtronic, Inc., Minneapolis, MN, USA), so two to three Nanostim devices (St. Jude Medical, Inc., St. Paul, MN, USA) should also be reasonable. Additionally, there may be an option for retrieving a chronic device, as demonstrated by late Nanostim (St. Jude Medical, Inc., St. Paul, MN, USA) retrievals.^14^ Technology is expected to continue to advance and likely will result in smaller devices; longer-lasting batteries; increased capabilities for AV synchrony, ventricular resynchronization, and/or antitachycardia pacing; or entirely new approaches in the future.

Now that leadless pacemaker implantation can be performed and a device (Micra; Medtronic, Inc., Minneapolis, MN, USA) is available that is both FDA-approved and covered by Medicare, who should be considered as appropriate candidates in current clinical practice? In the clinical trials, patients were enrolled who were suitable for ventricular (VVI) pacing, including those with: (1) permanent atrial fibrillation with AV block and bradycardia; (2) sinus rhythm with high-grade AV block and a limited expected level of physical activity or lifespan; or (3) sinus bradycardia with infrequent prolonged pauses, syncope, or His-Purkinje disease. These patients were not expected to require frequent ventricular pacing, as pacemaker dependence was an exclusion criterion. Additional exclusion criteria included patients with pulmonary hypertension, a mechanical tricuspid valve, an inferior vena cava filter, or pre-existing transvenous pacing or defibrillator leads. Other potential candidates in current practice might be patients with difficult upper body anatomy or vascular access issues and/or those who are not optimal candidates for transvenous systems, including those with prior pacing lead infections. In conclusion, the leadless cardiac pacemaker represents an important step forward in pacemaker technology, changing the nature of both the short- and long-term risks. As the technology advances, it will become increasingly important to consider the potential role for this device when assessing patients who need a pacemaker implant.

Ashish A. Bhimani, MD

ashish.bhimani@gmail.com

Cardiac Electrophysiologist

Piedmont Heart Institute

Atlanta, GA 30309

Bruce S. Stambler, MD

bss4@case.edu

Cardiac Electrophysiologist

Piedmont Heart Institute

Atlanta, GA 30309
